# Advances in the First Line Treatment of Pediatric Acute Myeloid Leukemia in the Polish Pediatric Leukemia and Lymphoma Study Group from 1983 to 2019

**DOI:** 10.3390/cancers13184536

**Published:** 2021-09-09

**Authors:** Małgorzata Czogała, Walentyna Balwierz, Katarzyna Pawińska-Wąsikowska, Teofila Książek, Karolina Bukowska-Strakova, Wojciech Czogała, Barbara Sikorska-Fic, Michał Matysiak, Jolanta Skalska-Sadowska, Jacek Wachowiak, Małgorzata Moj-Hackemer, Krzysztof Kałwak, Katarzyna Muszyńska-Rosłan, Maryna Krawczuk-Rybak, Dominik Grabowski, Jerzy Kowalczyk, Lucyna Maciejka-Kembłowska, Ninela Irga-Jaworska, Katarzyna Bobeff, Wojciech Młynarski, Renata Tomaszewska, Tomasz Szczepański, Agnieszka Chodała-Grzywacz, Grażyna Karolczyk, Agnieszka Mizia-Malarz, Katarzyna Mycko, Wanda Badowska, Karolina Zielezińska, Tomasz Urasiński, Justyna Urbańska-Rakus, Małgorzata Ciebiera, Radosław Chaber, Natalia Bartoszewicz, Mariusz Wysocki, Szymon Skoczeń

**Affiliations:** 1 Department of Pediatric Oncology and Hematology, Institute of Pediatrics, Jagiellonian University Medical College, 31-663 Krakow, Poland; malgorzata.czogala@uj.edu.pl (M.C.); walentyna.balwierz@uj.edu.pl (W.B.); katarzyna.pawinska-wasikowska@uj.edu.pl (K.P.-W.); 2 Department of Pediatric Oncology and Hematology, University Children Hospital, 30-663 Krakow, Poland; czogala@tlen.pl; 3 Department of Medical Genetics, Institute of Pediatrics, Jagiellonian University Medical College, 31-663 Krakow, Poland; teofilaksiazek@o2.pl; 4 Department of Clinical Immunology, Institute of Pediatrics, Jagiellonian University Medical College, 31-663 Krakow, Poland; k.bukowska-strakova@uj.edu.pl; 5 Department of Oncology, Pediatric Hematology, Transplantology and Pediatrics, Medical University of Warsaw, 02-091 Warsaw, Poland; basiasf@poczta.onet.pl (B.S.-F.); michal.matysiak@litewska.edu.pl (M.M.); 6 Department of Pediatric Oncology, Hematology and Transplantology, Poznan University of Medical Sciences, 60-572 Poznan, Poland; jsk@poczta.onet.eu (J.S.-S.); jacek.wachowiak@plusnet.pl (J.W.); 7 Department of Bone Marrow Transplantation, Pediatric Oncology and Hematology, Wroclaw Medical University, 50-556 Wrocław, Poland; malgorzata.moj@o2.pl (M.M.-H.); krzysztof.kalwak@umed.wroc.pl (K.K.); 8 Department of Pediatric Oncology and Hematology, Medical University of Bialystok, 15-276 Bialystok, Poland; kmroslan@umb.edu.pl (K.M.-R.); rybak@umb.edu.pl (M.K.-R.); 9 Department of Pediatric Hematology, Oncology and Transplantology, Medical University of Lublin, 20-090 Lublin, Poland; michaldominik@onet.pl (D.G.); jkowalcz@dsk.lublin.pl (J.K.); 10 Department of Pediatrics, Hematology and Oncology, Medical University of Gdansk, 80-211 Gdansk, Poland; lucynamaciejka@gumed.edu.pl (L.M.-K.); nirga@gumed.edu.pl (N.I.-J.); 11 Department of Pediatrics, Oncology and Hematology, Medical University of Lodz, 91-738 Lodz, Poland; katarzyna.bobeff@umed.lodz.pl (K.B.); wojciech.mlynarski@umed.lodz.pl (W.M.); 12 Department of Pediatrics Hematology and Oncology, Medical University of Silesia, 41-800 Zabrze, Poland; rtomaszewska@szpital.zabrze.pl (R.T.); szczep57@poczta.onet.pl (T.S.); 13 Department of Pediatric Hematology and Oncology, Regional Polyclinic Hospital in Kielce, 25-736 Kielce, Poland; aga.chodala@vp.pl (A.C.-G.); grazyna.karolczyk@wszzkielce.pl (G.K.); 14 Department of Oncology, Hematology and Chemotherapy, Upper Silesia Children’s Care Health Centre, Medical University of Silesia, 40-752 Katowice, Poland; amizia-malarz@sum.edu.pl; 15 Department of Pediatrics and Hematology and Oncology, Province Children’s Hospital, 10-561 Olsztyn, Poland; katarzynamycko@o2.pl (K.M.); hematologia@wssd.olsztyn.pl (W.B.); 16 Department of Pediatrics, Hematology and Oncology, Pomeranian Medical University, 71-252 Szczecin, Poland; karolina_zielezinska@wp.pl (K.Z.); urasin@pum.edu.pl (T.U.); 17 Department of Pediatrics, Hematology and Oncology, City Hospital, 41-500 Chorzow, Poland; jrakus@zsm.com.pl; 18 Department of Pediatric Oncohematology, Clinical Province Hospital of Rzeszow, 35-301 Rzeszów, Poland; onkohematologia.dzieci@szpital2.rzeszow.pl (M.C.); rchaber@wp.pl (R.C.); 19 Department of Pediatrics, Institute of Medical Sciences, Medical College, University of Rzeszow, 35-310 Rzeszow, Poland; 20 Department of Paediatrics, Haematology and Oncology, Nicolaus Copernicus University in Toruń Collegium Medicum in Bydgoszcz, 85-094 Bydgoszcz, Poland; natalabar@wp.pl (N.B.); m.wysocki@cm.umk.pl (M.W.)

**Keywords:** pediatric acute myeloid leukemia, survival, management

## Abstract

**Simple Summary:**

We retrospectively analyzed the results of the five consecutive treatment protocols for pediatric acute myeloid leukemia (AML) used in Poland from 1983 to 2019 (excluding promyelocytic, secondary, biphenotypic, and Down syndrome AML). The study included 899 children. The probability of three-year overall, event-free, and relapse-free survival increased from 0.34 ± 0.03 to 0.75 ± 0.05, 0.31 ± 0.03 to 0.67 ± 0.05, and 0.52 ± 0.03 to 0.78 ± 0.05, respectively. A systematic reduction of early deaths and deaths in remission was achieved, while the percentage of relapses decreased only in the last therapeutic period. Surprisingly good results were obtained in the group of patients with unfavorable genetic abnormalities like KMT2A-MLLT10/t(10;11)(p12;q23) and DEK-NUP214/t(6;9)(p23;q24) who were treated in the AML-BFM 2012 Registry, while an unsatisfactory outcome was found in patients with FLT3-ITD. The use of standardized therapeutic protocols with the successive consideration of genetic prognostic factors and advances in supportive care led to a significant improvement in AML treatment outcomes over the last 40 years.

**Abstract:**

Background: From 1983, standardized therapeutic protocols for pediatric acute myeloid leukemia (AML) based on the BFM group experience were introduced in Poland. We retrospectively analyzed the results of pediatric AML treatment in Poland from 1983 to 2019 (excluding promyelocytic, therapy-related, biphenotypic, and Down syndrome AML). Methods: The study included 899 children suffering from AML treated with the following: AML-PPPLBC 83 (1983–1993, *n* = 187), AML-PPGLBC 94 (1994–1997, *n* = 74), AML-PPGLBC 98 (1998–2004, *n* = 151), AML-BFM 2004 Interim (2004–2015, *n* = 356), and AML-BFM 2012 (2015–2019, *n* = 131). Results: The probability of three-year overall survival was 0.34 ± 0.03, 0.37 ± 0.05, 0.54 ± 0.04, 0.67 ± 0.03, and 0.75 ± 0.05; event-free survival was 0.31 ± 0.03, 0.34 ± 0.05, 0.44 ± 0.04, 0.53 ± 0.03, and 0.67 ± 0.05; and relapse-free survival was 0.52 ± 0.03, 0.65 ± 0.05, 0.58 ± 0.04, 0.66 ± 0.03, and 0.78 ± 0.05, respectively, in the subsequent periods. A systematic reduction of early deaths and deaths in remission was achieved, while the percentage of relapses decreased only in the last therapeutic period. Surprisingly good results were obtained in the group of patients treated with AML-BFM 2012 with unfavorable genetic abnormalities like KMT2A-MLLT10/t(10;11)(p12;q23) and DEK-NUP214/t(6;9)(p23;q24), while unsatisfactory outcomes were found in the patients with FLT3-ITD. Conclusions: The use of standardized, systematically modified therapeutic protocols, with the successive consideration of genetic prognostic factors, and advances in supportive care led to a significant improvement in AML treatment outcomes over the last 40 years.

## 1. Introduction

Acute myeloid leukemia (AML) is a heterogeneous disease with a clonal expansion of myeloid progenitors in the bone marrow and peripheral blood. It comprises about 15% of all leukemias in children. The treatment results in pediatric AML have improved significantly over the last 40 years, and the current overall survival is about 70% [1,2]. A history of unified therapeutic pediatric AML protocols in Poland began in 1983. The Polish Pediatric Leukemia/Lymphoma Study Group (PPLLSG) introduced protocol AML-PPLLSG 83, a modified version of Berlin–Frankfurt–Munster (BFM)-AML 83 protocol. The main aim of PPLLSG was to develop diagnostic and treatment standards, and to trial the utility of intensive protocols in Polish conditions. The protocol allowed for increasing the five-year EFS to 32% compared with less than 15% before 1983 [3,4]. However, the results were still significantly worse than the original BFM results [3,5]. The main cause of the difference was a high percentage of early deaths, resulting in a significantly lower remission rate. Insufficient supportive care and toxicities were the main restriction in the introduction of high doses of cytarabine in the consecutive protocols. In the new protocol (AML-PPLLSG 94) stratification into risk groups was implemented. This caused a slight, but not significant, increase in the event-free survival (EFS) rate (from 32 to 36%) [3,4]. However, the ID-ARAC courses were not effective at producing morphological remissions in late responders and the remission rate did not increase [3]. In the consecutive protocol, AML-PPLLSG 98, a more accurate stratification system was implemented and a new drug, idarubicine, was introduced instead of daunorubicine, for the induction, intensification, and consolidation. Better initial responses were observed in the SR group; however, unsatisfactory treatment outcomes were found in the HR group [3,4].

In 2005, the AML-BFM 2004 Interim protocol was introduced in PPLLSG centers. The stratification to the risk groups was based on the FAB classification, genetic abnormalities, and response to the treatment. Since that time, the central verification of the cell morphology, immunophenotyping, and cytogenetic examination has been available. Since 2006, molecular studies (fusion genes AML1-ETO, PML-RARA, CBFβ-MYH11, MLL-AF4, MLL-AF9, and MLL-ENL, as well as FLT3-ITD and WT1 overexpression) have been performed in the central laboratory in Krakow as the standard diagnostic procedure in all pediatric patients with AML. Allogeneic hematopoietic stem cell transplantation (allo-HSCT) from HLA-matched sibling donors was introduced for all patients of the HR group in the first morphological remission, and allo-HSCT from an HLA-matched unrelated donor for children with persistent blasts after the second induction (HAM) or still in aplasia 4 weeks after the second induction. A further improvement of the treatment results, an increase in the proportion of achieved remission, and a reduction of early deaths and deaths in remission was observed; however, the proportion of relapses was still high.

In 2015, a new treatment protocol AML-BFM 2012 was introduced. Stratification to the risk groups was based on cytogenetic and molecular genetics changes. The molecular diagnostic is extended by the HemaVision kit and an analysis of the additional gene mutations (NPM1 and CEBPα). Three risk groups were defined. Cladribine was added for the intermediate and high-risk group in the third chemotherapy course. Hematopoietic stem cell transplantation was indicated in all patients in the high-risk group. A further improvement of the treatment results was achieved.

In parallel with the therapeutic protocol improvements, supportive care also developed, including antifungal prophylaxis, broad spectrum antibiotic in case of neutropenic fever, and guidelines for emergencies like hyperleukocytosis and tumor lysis syndrome.

This is retrospective analysis of the results of the five consecutive treatment protocols for pediatric AML used in Poland from 1983 to 2019.

## 2. Materials and Methods

From 1983 to June 2019, there were 1225 patients from 0 to 18 years old with novo AML diagnosed in Poland. We excluded patients with secondary AML (40), acute promyelocytic leukemia (98), biphenotypic leukemia (23), and patients with Down syndrome myeloid leukemia (68). In addition, 17 patients died before the beginning of the treatment, 15 patients were pretreated with another protocol because of wrong initial diagnosis (acute lymphoblastic leukemia, neuroblastoma), 5 patients could not follow the treatment protocol because of coexisting morbidities, and 16 patients were excluded because of the lack of data. Finally, 899 patients were enrolled in the retrospective analysis. The data were collected through the Polish Acute Myeloid Leukemia Registry.

The patient characteristics are presented in the Table 1.

The patients were treated in 16 Polish pediatric oncology and hematology centers associated with PPLLSG. Five consecutive protocols were used, namely: period I (1983−1993, protocol AML-PPLLSG, 187 patients), period II (1994−1998, AML-PPLLSG 94, 74 patients), period III (1998−2004, AML-PPLLSG 98, 151 patients), period IV (2004−2015, AML-BFM 2004 Interim, 356 patients), and period V (2015−2019, AML-BFM 2012 Registry, 131 patients).

**Figure 1:**
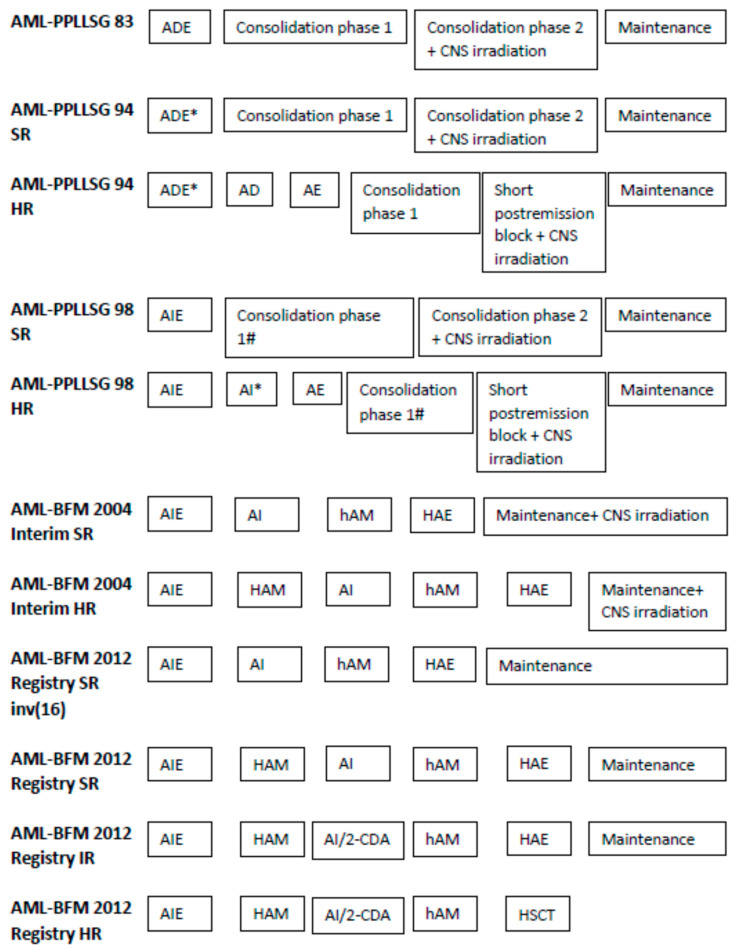
Treatment protocols. ADE: cytarabine 100 mg/m^2^/day continuous infusion on days 1 and 2, followed by 30 min infusion every 12 h on days 3–8; daunorubicin 60 mg/m^2^, 30 min infusion on days 3–5; and etoposide 150 mg/m^2^, 60 min infusion on days 6–8. Consolidation phase 1: 6-thioguanine 60 mg/m^2^/day, days 1–28, orally; prednisone 60 mg/m^2^/day, days 1–28, orally; cytarabine 75 mg/m^2^/day, days 3–6, 10–13, 17–20, 2and 4–27, i.v. or s.c.; vincristine 1.5 mg/m^2^/day, days 1, 8, 15, and 22; doxorubicin 30 mg/m^2^/day, days 1, 8, 15, and 22. Consolidation phase 2: 6-thioguanine 60 mg/m^2^/day, days 29–57 orally; cytarabine 75 mg/m^2^/day, days 31–34, 38–41, 45–48, and 52–55, i.v or s.c.; cyclophosphamide 500 mg/m^2^/day, 60 min infusion on days 29 and 57. ADE*: cytarabine 100 mg/m^2^/day continuous infusion on days 1 and 2, followed by 30 min infusion every 12 h on days 3–8; daunorubicin 30 mg/m^2^/dose, 30 min infusion every 12 h on days 3, 4, and 5; etoposide 150 mg/m^2^/day, 60 min infusion on days 6–8. AD: cytarabine 500 mg/m^2^/dose, 60 min infusion every 12 h on days 1–5 (10 doses); daunorubicin 30 mg/m^2^/day, 30 min infusion on days 1, 3, and 5. AE: cytarabine 500 mg/m^2^/dose, 60 min infusion every 12 h on days 1–5 (10 doses); etoposide 100 mg/m^2^/day, 60 min infusion on days 1–5. Short post-remission block: 6-thioguanine 60 mg/m^2^/day on days 1–15, orally; cytarabine 75 mg/m^2^/day on days 3–6, 10–13, i.v. or s.c.; cyclophosphamide 500 mg/m^2^/day, 60 min infusion on day 1. AIE: cytarabine 100 mg/m^2^/day continuous infusion on days 1 and 2, followed by 30 min infusion every 12 h on days 3–8; idarubicin 12 mg/m^2^/day, 30 min infusion days 3, 4, and 5; etoposide 150 mg/m^2^/day, 60 min infusion on days 6–8. Consolidation phase 1#: 6-thioguanine 60 mg/m^2^/day, days 1–28, orally; prednisone 60 mg/m^2^/day, days 1–28, orally; cytarabine 75 mg/m^2^/day, days 3–6, 10–13, 17–20, and 24–27, i.v. or s.c.; vincristine 1.5 mg/m^2^/day, days 1, 8, 15, and 22; idarubicin 12 mg/m^2^/day, 30 min infusion on days 1, 8, 15, and 22. AI*: cytarabine 500 mg/m^2^/dose, 60 min infusion every 12 h on days 1–5 (10 doses); idarubicin 12 mg/m^2^/day, 30 min infusion on days 1, 3, and 5. AI: cytarabine 500 mg/m^2^/day, 96-hours infusion on days 1–4; idarubicin 12 mg/m^2^/day, 30 min infusion on days 3 and 5. hAM: cytarabine 1 g/m^2^/dose, 3 h infusion every 12 h on days 1–3 (6 doses); mitoxantrone 10 mg/m^2^/day, 30min infusion on days 3 and 4. HAE: cytarabine 3 g/m^2^/dose, 3 h infusion every 12 h on days 1–3 (6 doses); etoposide 125 mg/m^2^/day, 60 min infusion on days 2–5. HAM: cytarabine 3 g/m^2^/dose, 3 h infusion every 12 h on days 1–3 (6 doses); mitoxantrone 10 mg/m^2^/day, 30 min infusion on days 3 and 4. AI/2-CDA: cytarabine 500 mg/m^2^/day, 96-h infusion on days 1–4; idarubicin 12 mg/m^2^/day, 30 min infusion on days 3 and 5; 2-chloro-2-deoxyadenosine 6 mg/m^2^/day, 30 min infusion, on days 1 and 3. Maintenance: AML-PPLLSG 83: Daily thioguanine 40 mg/m^2^/day, orally; cytarabine 40 mg/m^2^/day, i.v. or s.c. for 4 days every 4 weeks for 2 years; doxorubicin 25 mg/m^2^/day every 8 weeks during the first year. AML-PPLLSG 94 and 98: Daily thioguanine 40 mg/m^2^/day, orally; cytarabine 40 mg/m^2^/day, i.v. or s.c., 4 days every 4 weeks—SR groups for up to 2 years, HR groups received therapy for 1 year (about 18 months of therapy together with intensive courses). AML-BFM 2004 and AML-BFM 2012: daily thioguanine 40 mg/m^2^/day, orally; cytarabine 40 mg/m^2^/day, i.v. or s.c., 4 days every 4 weeks for 1 year.

Details of the treatment are presented in Figure 1 and Table 2. Table 3 contains stratification to the risk groups in the periods II−V. Indications for HSCT and the number of patients who underwent the procedure are shown in Table 4.

CSN prophylaxis and therapy are presented in Table 2.

### 2.1. Diagnostics

The diagnosis was based on bone marrow (BM) examination, including morphology with FAB classification and immunophenotyping. Cytogenetic analyses (both classical and fluorescence in situ hybridization (FISH)) performed in local treatment centers were mandatory from 1998. The results of the karyotype were eligible in 449 patients, including 67 (44%), 267 (75%), and 115 (88%) from periods III, IV, and V, respectively. Since 2005 (period IV and V), central verification of the cell morphology and immunophenotyping examination has been introduced. Moreover, since 2006, molecular studies (fusion genes AML1-ETO, PML-RARA, CBFβ-MYH11, KMT2A-MLLT1, KMT2A–MLLT3, and KMT2A–AFF1) have also been performed in the central Genetic Laboratory of the University Children’s Hospital of Krakow. The results are available for 292 patients from period IV (82%). Since 2015 (period V), the molecular diagnostics has been extended by the HemaVision kit and through analysis of additional gene mutations (NPM1 and CEBPα). These results are available for 125 patients for period V (95%). Analysis of the FLT3-ITD was performed from 2005, and the results for 410 patients are available (278 from period IV (78%) and 129 from period V (100%)).

The data concerning the cytogenetics and molecular genetics of the patients from periods IV and V are presented in Table 5.

### 2.2. Definitions

Complete morphological remission is defined as less than 5% of blasts in BM demonstrating normal or only slightly decreased cellularity, with signs of regeneration of normal hematopoiesis, regeneration of normal cell production in peripheral blood (≥1000/μL leukocytes, ≥500/μL neutrophil granulocytes and ≥50,000/μL platelets), lack of blasts in peripheral blood, and the disappearance of any extramedullary sites. Minimal residual disease was assessed only in the last period as a part of a separate study; these results did not influence the therapeutic decision and were not analyzed in that paper.

Treatment failure included early death (death during the first 42 days from the start of the treatment); failure to achieve morphological remission on the first line therapy (no fulfillment of CR criteria by the end of intensification; leukemic blasts ≥5% after the second induction; aplasia ≥6 weeks after the beginning of the second induction) and relapse (the reappearance of leukemic blasts in the peripheral blood; re-infiltration of BM with >5% distinct blasts not attributable to any other cause; leukemic infiltration elsewhere following CR; or partial remission lasting at least 4 weeks).

Statistical analyses were performed with STATISTICA, version 13, StatSoft Inc. Probabilities of survival were calculated using the Kaplan−Meier method. Overall survival (OS) was defined as the time from the date of diagnosis to the date of death from any cause, or from the last follow-up. Event-free survival (EFS) was calculated as the time from diagnosis to the first event (relapse, death of any cause, failure to achieve remission, or secondary malignancy) or last follow-up. Failure to achieve morphological remission was considered an event on day 0. Relapse-free survival (RFS) was calculated as the time from the first remission to the relapse. Probabilities of survival were presented as decimal fractions with standard deviations. The sub-groups were compared with a log-rank test. The relative risks were estimated using a Cox proportional hazard model. The results are presented as a hazard ratio (HR) with a 95% confidence interval (CI). All variables having a *p* value <0.05 in the univariate analysis were included in a multivariate analysis.

The study was conducted according to the guidelines of the Declaration of Helsinki, and was approved by the Ethics Committee of Jagiellonian University (protocol code: 122.6120.17.2015, date of approval: 29 January 2015). Informed consent was obtained from all patients or guardians when entering each therapeutic protocol.

## 3. Results

### 3.1. Treatment Results

**Figure 2:**
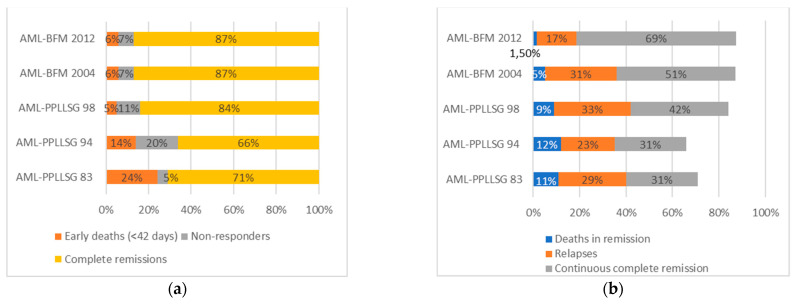
Treatment results. (**a**) Percentages of early deaths, non-responders, achieved complete morphological remissions in the five consecutive periods. (**b**) Percentages of deaths in remission, relapses, and patients in continuous complete morphological remission in the five consecutive periods.

The percentage of achieved remissions increased from 72% in period I to 87% in periods IV and V. First-line non-responders comprised 6%, 18%, 10%, 8%, and 7%, respectively. A reduction of early deaths (up to the 42nd day of treatment) was noted, and the percentage decreased systematically in periods I−V from 22% to 5%. Among the patients who achieved remission, the percentage of deaths from toxicities in the following periods decreased from 16% to 1.6%. The rates of relapse were similar in periods I−IV (about 30%) and decreased in the last period to 17% (median follow-up in period V of 37 months; Table 1). The results are presented in Figure 2 and Table 6.

**Figure 3:**
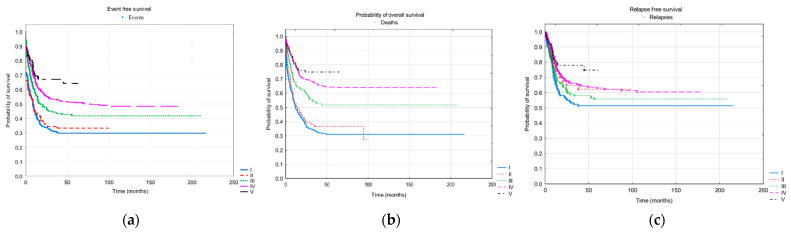
Probability of survival in the consecutive periods: (I) AML-PPLLSG 83, (II) AML-PPLLSG 94, (III) AML-PPLLSG 98, (IV) AML-BFM 2004 Interim, (V) AML-BFM 2012 Registry. Statistically significant differences (log-rank test) are marked. (**a**) Probabilities of three-year overall survival ± standard deviations (SD): 0.34 ± 0.03, 0.37 ± 0.05, 0.54 ± 0.04, 0.67 ± 0.03, and 0.75 ± 0.05 in periods I−V, respectively. Statistically significant differences (log-rank test) between periods I and III (*p* = 0.0003), IV (*p* < 0.000001), and V (*p* < 0.000001); II and III (0.01129), IV (*p* < 0.000001), and V (*p* < 0.000001); III and IV (0.00661) and V (*p* = 0.00076). (**b**) Probabilities of three-year event free survival ± SD: 0.31 ± 0.03, 0.34 ± 0.05, 0.44 ± 0.04, 0.53 ± 0.03, and 0.67 ± 0.05 in periods I−V, respectively. Statistically significant differences (log-rank test) between periods I and III (*p* = 0.00518), IV (*p* < 0.00001), and V (*p* < 0.00001); II and IV (*p* = 0.00117) and V (*p* < 0.00001). (**c**) Probabilities of three-year relapse-free survival counted for patients who achieved morphological remission ± SD: 0.52 ± 0.03, 0.65 ± 0.05, 0.58 ± 0.04, 0.66 ± 0.03, and 0.78 ± 0.05 in periods I–V. Statistically significant differences (log-rank test) between periods I and IV (*p* = 0.03464) and V (*p* = 0.00103), and IV and V (*p* = 0.01403).

In the subsequent periods, survival improved significantly. The probability of three-year OS increased from 0.34 ± 0.03 to 0.75 ± 0.05, EFS from 0.31 ± 0.03 to 0.67 ± 0.05, and RFS from 0.52 ± 0.03 to 0.78 ± 0.05. The survival curves are presented in Figure 3, and the probabilities of survival are in Table 6.

### 3.2. Risk Groups

The outcome within the risk groups was analyzed in the last two periods. A comparison of the survival between risk groups was performed separately for each period, as the definition of the risk groups differed across the therapeutic protocols.

According to the AML-BFM 2004 protocol (period IV), two risk groups were distinguished. There were 71 patients (19.9%) in the SRG and 284 children (79.8%) in the HRG (no data in 1 patient). The probability of five-year OS was significantly higher in SRG (0.74 ± 0.04) compared with HRG (0.62 ± 0.03; p = 0.03178). The difference in the probability of EFS was almost significant between SRG and HRG (0.56 ± 0.04 vs. 0.47 ± 0.03; *p* = 0.0567). No significant differences were found in the probability of RFS (SRG 0.59 ± 0.04, HRG 0.61 ± 0.03). The survival curves are shown in Figure S1.

In period V, three risk groups were defined. There were 25 children in SRG (18.5%), 52 patients in IRG (40%), and 52 in HGR (40%); no data were available concerning two patients. The probabilities of five-year OS were 0.92 ± 0.08, 0.81 ± 0.06, and 0.64 ± 0.06 in SRG, IRG, and HRG, respectively. There was a significant difference in OS between SRG and HRG (*p* = 0.01296). The probability of five-year EFS was significantly higher in SRG compared with HRG (0.83 ± 0.08 vs. 0.53 ± 0.06; *p* = 0.01925), with no difference in comparison to IRG (0.69 ± 0.06). No significant difference between the risk groups was noted in terms of RFS (0.83 ± 0.9, 0.71 ± 0.06, and 0.74 ± 0.07 in SRG, IRG, and HRG, respectively). Survival curves are presented in Figure S2.

### 3.3. The Treatment Results Depending on the Genetics

#### 3.3.1. FLT3-ITD

The analysis was performed only for patients treated with AML-BFM 2004 (period IV) and AML-BFM 2012 (period V) as the results of the FLT3-ITD analysis were not available for the patients from periods I−III. There were 41 FLT3-ITD positive (30 in period IV and 11 in period V) and 366 negative patients (248 in period IV and 118 in he period V).

The probabilities of OS, EFS, and RFS were significantly lower in the FLT3-ITD positive patients compared with the FLT3-ITD negative patients (OS: 0.49 ± 0.08 vs. 0.70 ± 0.02, *p* = 0.0137; EFS: 0.31 ± 0.07 vs. 0.58 ± 0.03, *p* = 0.00014; RFS: 0.41 ± 0.09 vs. 0.68 ± 0.03, *p* = 0.00078; Figure S3). There were no significant differences in OS, EFS, and RFS in the FLT3-ITD positive patients, depending on the treatment protocol.

#### 3.3.2. Fusion Genes

The results of CBFB-MYH11 and RUNX1-RUNX1T1 were available for most patients treated with AML-BFM 2004 (period IV) and AML-BFM 2012 (period V). There were 24 patients (6%) with the CBFB-MYH11 fusion gene (12 in period IV and 12 in period V). The probabilities of five-year OS, EFS, and RFS were 0.82 ± 0.08, 0.65 ± 0.11, and 0.74 ± 0.11, respectively. The survival curves are presented in Figure S4. No resistant disease was noted in that group of patients. In period IV, there were four relapses and three patients died (all because of toxicities, including one after HSCT in the second remission). In period V, there were no relapses, and one patient died in remission because of severe infection. No significant differences were found in the probabilities of survival between patients treated according to AML-BFM 2004 and AML-BFM 2012 (OS 0.75 ± 0.12 vs. 0.92 ± 0.12, *p* = 0.41516; EFS 0.50 ± 0.12 vs. 0.92 ± 0.12, *p* = 0.10814; RFS 0.60 ± 0.13 vs. 1.0 ± 0.13, *p* = 0.09919).

The RUNX1-RUNX1T1 fusion gene was identified in 66 patients (16%) in the analyzed group (51 in the period IV and 11 in the period V). Only one patient (treated with AML-BFM 2004) did not achieve morphological remission and died in the course of disease progression. Twenty children relapsed (16/51 (31%) in period IV and 4/16 (25%) in period V), including three patients with two relapses (one of them died because of the disease progression). In total, 13 children died—2 of progression (1 non-responder and 1 after second relapse) and 11 of toxicities (4 in the I CR and 7 in the II CR, including 4 after HSCT). All of the deaths occurred in period IV. The probabilities of five-year OS, EFS, and RFS were 0.79 ± 0.05, 0.62 ± 0.06, and 0.68 ± 0.06, respectively. Survival curves are shown in Figure S5. Probability of OS was almost significantly higher in period V compared with period IV (1.0 vs. 0.74, *p* = 0.05239), and no significant differences were found in the probability of EFS and RFS between periods IV and V (0.61 ± 0.07 vs. 0.71 ± 0.1 and 0.67 ± 0.08 vs. 0.71 ± 0.12; Figure S6).

**Figure 4:**
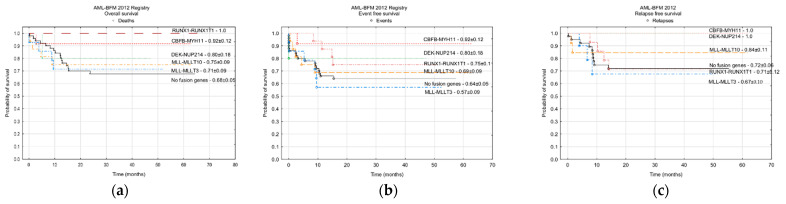
Probability of survival depending on the fusion genes in patients treated with the AML-BFM 2012 Registry. Probabilities of five-year survival are shown: (**a**) overall survival; (**b**) event free survival; (**c**) relapse free survival.

An analysis of survival depending on the other fusions genes was performed only for the patients from period V. In that group, the HemaVision test including 28 fusion genes was used. The results were available for 125 children (95%). Survival curves were done for the fusion genes found in at least five patients, including: CBFB-MYH11 (12 children), RUNX1-RUNX1T1 (16), KMT2A-MLLT10 (16), KMT2A-MLLT3 (14), DEK-NUP214 (5), and for the group of 52 patients with no fusion gene identified with HemaVision. The highest probability of OS was noted for the patients with RUNX1-RUNX1T1 (no deaths), and was significantly higher than in the patients with MLL-MLLT3, MLL-MLLT10, and children with no fusion genes (Figure 4). No other significant differences in OS were found. Patients with CBFB-MYH11 tended to have the highest probability of EFS and RFS. Almost significant differences in the EFS and RFS were noted between children with CBFB-MYH11 and patients with MLL-MLLT3 and without fusion genes (Figure 4).

### 3.4. Prognostic Factors—Cox Regression Modelling

To assess the significance of potential prognostic factors, Cox regression modelling was performed. The univariate analysis comprised age as the quantitative and qualitative (<2 years, 2–10 years and >10 years) variables, number of white blood cells (WBC) at diagnosis as the quantitative and qualitative (<20,000/µL, 20,000/µL–100,000/µL, and >100,000/µL) variables, and the most common fusion genes or mutations (CBFβ-MYH11, RUNX1-RUNX1T1, MLLL-MLLT10, and MLL-MLLT3,FLT3-ITD and WT1) for OS, EFS, and RFS. It revealed that the WT1 mutation, FLT3-ITD, and WBC >100,000/µL at diagnosis predicted a poor outcome, while RUNX1-RUNX1T1 was a favorable prognostic factor. The results are presented in Table 7. In the multivariate analysis, only WBC at diagnosis >100,000/µL remained a significant predictor of poor outcomes (HR for EFS 2.32, 95%CI: 1.20–4.48, *p* = 0.012263; HR for OS 2.75, 95%CI 1.28–5.94, *p* = 0.009764).

### 3.5. Hematopoietic Stem Cell Transplantation

In the whole analyzed cohort, 173 patients received HSCT in the first CR. As the number of transplantations in the first two periods was low and the data concerning HSCT from that time were lacking, the next analysis considered the children from periods III−V. During that time, HSCT was performed in 157 patients, including 20 in period III, 100 in period IV, and 37 in period V. There were 10 auto-HSCT and 147 allo-HSCT (Table 4).

**Figure 5:**
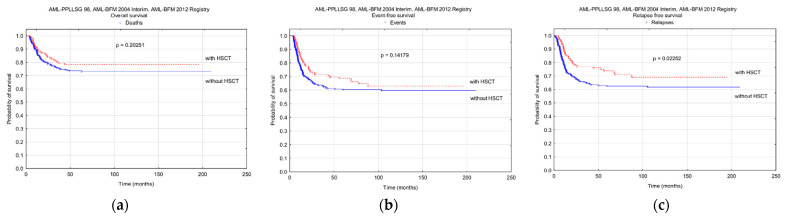
Probability of survival in patients treated with AML-PPLLSG 98, AML-BFM 2004 Interim, and AML-BFM 2012 Registry with and without hematopoietic stem cell transplantation (HSCT). The patients with event-free survival shorter than 4 months were excluded from the analysis: (**a**) overall survival; (**b**) event free survival; (**c**) relapse free survival.

An analysis of the survival was performed for the patients with event-free survival longer than 4 months (HSCT was performed after at least four chemotherapy cycles, which means after at least 4 months from diagnosis). There was a significant difference in RFS between patients with and without HSCT (five-year RFS 0.74 ± 0.04 vs. 0.62 ± 0.03, *p* = 0.02252), while there were no differences in OS and EFS (five-year OS 0.79 ± 0.03 vs. 0.74 ± 0.02, *p* = 0.20251; EFS 0.69 ± 0.03 vs. 0.60 ± 0.02, *p* = 0.14179). Survival curves are shown in Figure 5.

In total, 37/157 patients (27%) died after HSCT in the analyzed period, including 4/20 (20%) patients treated with AML-PPLLSG 98, 26/100 (26%) children treated with AML-BFM 2004, and 7/37 (19%) patients treated with AML-BFM 2012. There were 16 deaths of toxicities after HSCT (10% of all transplanted patient), including 2 (10%), 11 (11%), and 3 (8%) patients from periods III, IV, and V, respectively. Twenty patients (13%) died of progressive disease after HSCT, including 2 (10%), 15 (15%) and 3 (8%) patients treated with consecutive protocols (periods III−V). No significant differences were found in OS, EFS, and RFS in children treated with HSCT depending on the treatment protocols (five-year OS: 0.77 ± 0.11, 0.74 ± 0.03 and 0.80 ± 0.06; EFS: 0.49 ± 0.11, 0.58 ± 0.03, 0.65 ± 0.6; RFS: 0.49 ± 0.12; 0.71 ± 0.04; 0.75 ± 0.07, in periods III, IV, and V, respectively).

## 4. Discussion

The treatment results in childhood AML in Poland improved significantly during the last 40 years. Probability of OS, EFS, and RSF increased from 0.31 ± 0.03 to 0.75 ± 0.05, from 0.30 ± 0.03 to 0.65 ± 0.05, and from 0.51 ± 0.03 to 0.75 ± 0.05, respectively. At the beginning of the history of unified AML treatment protocols in Poland, a high number of early deaths was observed, leading to a lower percentage of morphological remission. In the protocol AML-PPLLSG 94 with intermediate doses of cytarabine, the percentage of early deaths was reduced; however, the non-responders rate increased. Improvement of the supportive care enabled further intensification of the treatment. Introducing idarubicine in protocol AML-PPLLSG 98 and high-dose cytarabine in AML-BFM 2004 led to a significant decrease of non-responders compared to earlier therapy, and a significant increase of morphological remission. This was consistent with the reports of the BFM Study Group [6,7]. Despite the improvement in the treatment outcome in periods I-IV, the percentage of relapses remained unsatisfactory high. A significant reduction of relapses was observed in the last period (AML-BFM 2012 Registry); however, the follow-up was much shorter (median 37 months) than in previous periods.

In addition to therapeutic protocol improvement, supportive care developed, including antifungal prophylaxis, broad spectrum antibiotic for neutropenic fever, and experience in management with emergencies like hyperleukocytosis or tumor lysis syndrome. All of this contributed to a reduction in early deaths and deaths in remission, and finally to an increase in overall survival. The significant role of supportive care in the improvement of the AML treatment outcome was reported by other authors [2,8,9,10,11].

Identification of the prognostic factors enabled stratification to the risk groups. At the beginning, it was based on the treatment response and FAB classification. Further studies of the AML-BFM Group, as well as clinical trials worldwide, revealed the prognostic significance of numerous genetic abnormalities [12,13,14,15,16,17,18]. Since introduction of the protocol AML-BFM 2004, genetic abnormalities were taken into account in the stratification to risk groups (t(8;21), inv(16) as a favorable and FLT3-ITD as adverse). In the protocol AML-BFM 2012, the genetic abnormalities became crucial as the prognostic factors (Table 3). In our study, an analysis of survival within risk groups was performed for the last two periods. In the group treated with AML-BFM 2004 Interim, the probability of five-year OS was significantly higher in the SRG compared to HRG. Difference in the probability of EFS was almost significant between SRG and HRG, while no significant differences were found in the probability of RFS. Similarly, in the protocol AML-BFM 2012 Registry there was a significant difference in the OS and EFS between SRG and HRG, while no significant difference between the risk groups was noted in terms of RFS. According to the risk group criteria, all non-responders were classified to the HRG, which could mainly affect lower EFS or OS in that group, while the HR patients who managed to achieve morphological remission had a similar risk of relapse as the SR patients. The relatively low rate of relapses in the HRG could be the effect of HSCT recommended for the high-risk patients.

Compared with the results of original AML-BFM 2004 protocol published by Creutzig [19] survival in the SRG in our group treated with AML-BFM 2004 Interim was lower (OS 0.89 ± 0.02 vs 0.74 ± 0.04, EFS 0.71 ± 0.03 vs 0.56 ± 0.04) while HRG outcome was similar (OS 0.65 ± 0.03 vs. 0.62 ± 0.03, EFS 0.46 ± 0.03 vs. 0.47 ± 0.03).

The outcome in the patients treated with AML-BFM 2012 in PPLLSG was comparable to the results reported by the AML-BFM Study Group (three-year OS 75 ± 0.05 vs. 0.82 ± 0.03 and three-year EFS 0.67 ± 0.05 vs. 0.69 ± 0.04, respectively) [20].

In our study, the results of the FLT3 analysis were available for 407 patients treated with AML-BFM 2004 and AML-BFM 2012, 41 of which (10%) were FLT3-ITD positive. That is similar to what has been reported by other authors [16,17]. In the Children’s Cancer Group (CCG) study, FLT3-ITD was present in 77 of the 360 tested patients (12%) [16], and in the study performed by the AML-BFM Study Group, this mutation was found in 52 out of the 353 analyzed patients (15%) [17]. In our cohort, the probabilities of OS, EFS, and RFS were significantly lower in the FLT3-ITD positive children compared with the FLT3-ITD negative patients. There were no significant differences in OS, EFS, and RFS in the FLT3-ITD positive patients depending on the treatment protocol (AML-BFM 2004 vs. AML-BFM 2012). The treatment results in the analyzed FLT3-ITD group were similar to the results reported by other authors. In the study published by Nicoreth et al. in the group of 52 FLT3-ITD positive patients treated in Germany with AML-BFM 2004 and AML-BFM 2012, the probability of three-year OS and EFS survival was 0.54 ± 0.07 and 0.39 ± 0.07, respectively [17]. In the CCG study, FLT3-ITD positive patients treated on CCG-2941 and -2961 between September 1995 and December 2001 had four-year OS of 0.33 ± 0.13 [16]. Targeted treatment with FLT3 inhibitors has been studied in the last two decades with promising results [1,21,22]. Midostaurin, one of the first-generation FLT3 inhibitors, is the first tyrosine kinase inhibitor approved by the FDA for AML therapy in the first line, and is now being investigated in combination with chemotherapy in children with newly diagnosed FLT3-mutated AML [23].

Inv(16)(p13q22) is associated with acute myeloid leukemia subtype M4Eo and is known as a favorable prognostic factor. It results in the fusion of CBFB and MYH11 genes. The rearrangement is described in about 8% of pediatric AML [13,24]. In our study, there were 24 patients (6% of children with available results) with the CBFB-MYH11 fusion gene (12 in period IV and 12 in period V). No resistant disease was noted in that group of patients. In period IV, there were four relapses and three patients died (all because of toxicities). In period V, there were no relapses, and one patient died in remission because of severe infection. Probabilities of five-year OS, EFS, and RFS were 0.82 ± 0.08, 0.65 ± 0.11 and 0.74 ± 0.11, respectively. The outcome in patients with inv(16) treated with AML-BFM 2012 seemed to be better compared with the protocol AML-BFM 2004; however, the differences were not statistically significant, probably because of the low number of patients in the subgroups. The results are similar to those reported by other authors [12,13].

Translocation (8;21) with fusion gene RUNX1-RUNX1T1 is well known as a favorable prognostic factor in AML, and is detected in 12–14% of pediatric AML [13,25]. In the protocol, AML-BFM 2004 patients with t(8;21) were classified to SRG, and were treated without the reinduction with high-dose cytarabine and mitoxantrone to minimize overtreatment. However, it was found that the reduction of the intensity of the treatment caused deterioration of outcome. [24]. Based on this finding, it was recommended to add a reinduction for the patients with t(8;21) in the consecutive protocol AML-BFM 2012. In our cohort, this translocation was found in 67 patients (16% of children with available results), including 51 patients treated with AML-BFM 2004 (period IV) and 16 children treated with AML-BFM 2012 (period V). The probabilities of five-year OS, EFS, and RFS were 0.79 ± 0.05, 0.62 ± 0.06, and 0.68 ± 0.06, respectively. Probability of OS was almost significantly higher in period V compared with period IV. However, despite the treatment intensification in the protocol AML-BFM 2012 (additional reinduction with HAM cycle), no reduction of relapses was noted. No significant differences were found in the probability of EFS and RFS between periods IV and V. Interestingly, the high rate of relapses did not deteriorate the OS. Patients who relapsed still had a favorable prognosis. Improvement in OS survival of patients with t(8;21) in period V compared with period IV was mainly due to a reduction of toxic deaths, probably as a result of better supportive care.

The complex analysis of fusion genes became a standard in the protocol AML-BFM 2012 (HemaVision test including 28 fusion genes). Probability of survival was assessed for the fusion genes found in at least five patients, including: CBFB-MYH11 (12 children), RUNX1-RUNX1T1 (16), KMT2A-MLLT10 (16), KMT2A-MLLT3 (14), DEK-NUP214 (5), and for the group of 52 patients with no fusion gene identified with HemaVision. The most favorable outcome was observed in patients with CBFβ-MYH11 and RUNX1-RUNX1T1 (despite the relatively low RFS in RUNX1-RUNX1T1), similarly to what has been reported by other authors [13,18]. Interestingly, outcome in patients with fusion genes known as adverse prognostic factors (KMT2A-MLLT10 and DEK-NUP214) was surprisingly good.

KMT2A-MLLT10/t(10;11)(p12;q23) was identified in almost 13% of patients in our cohort, while its occurrence is estimated as being 3% in large cohort studies [13,26]. Probability of five-year OS, EFS, and RFS in the group of patients with KMT2A -MLLT10 t(10;11)(p12;q23) in our research was 0.75 ± 0.09, 0.69 ± 0.09, and 0.84 ± 0.11, respectively. In comparison, in the study of Balgobind et al. involving 98 patients with t(10;11)(p12;q23) treated from 1993 to 2005 in 11 collaborative study groups, the probabilities of OS and EFS were 0.45 ± 0.05 and 0.31 ± 0.05, respectively. t(10;11)(p12;q23) were identified as independent predictors of an unfavorable prognosis [27]. The difference in outcome in that historical study compared with our results could be explained by fact that all patients in our study with that translocation were classified to the HRG with an indication to HSCT. However, HSCT was also performed in the cohort described by Balgobind et al., and this procedure did not predict the EFS or OS in the group of patients with t(10;11)(p12;q23) [27].

Translocation t(6;9)(p23;q24) with DEK-NUP214 was identified in five patients in our cohort. An excellent outcome was observed in that group with a probability of five-year OS, EFS, and RFS of 0.80 ± 0.18, 0.80 ± 0.18, and1.0 in contrast with the results reported by other authors. However, the number of patients with DEK-NUP214 was limited in our study. In the report from the Children’s Oncology Group assessing 48 patients with t(6;9), the probability of five-year OS, EFS, and disease free survival was 0.39 ± 0.15, 0.32 ± 0.14, and 0.33 ± 0.17%, respectively [28]. It was suggested in the literature that HSCT provides a survival benefit to patients with t(6;9) [28,29]. All five patients with DEK-NUP214 in our study were transplanted, which may explain the favorable outcome.

Allogeneic hematopoietic stem cell transplantation (allo-HSCT) has been shown to benefit patients with HR pediatric AML [1,30,31]. An improvement in supportive care led to a reduction of transplantation-related mortality in the last decades. In our cohort, 173 patients received HSCT in the first CR. Analysis of the survival concerning patients treated with AML-PPLLSG 98, AML-BFM 2004, and AML-BFM 2012 after exclusion of the patients with event-free survival shorter than 4 months revealed an improvement in the probability of RFS in the group with HSCT in the first CR compared with the patients treated without HSCT (five-year RFS 0.74 ± 0.04 vs. 0.62 ± 0.03, *p* = 0.02252). The percentage of death from toxicities and from the disease progression was 10% and 13%, respectively. The results are similar to those described by other authors [32,33].

## 5. Conclusions

The use of standardized, systematically modified therapeutic protocols, with successive consideration of genetic prognostic factors in stratification into risk groups, and advances in supportive care significantly improved AML treatment outcomes over the past 40 years. Currently, the treatment results in pediatric AML in Poland are comparable to the results described by other research groups. Surprisingly good results were obtained in the group of patients with genetic abnormalities known as adverse prognostic factors like KMT2A -MLLT10/t(10;11)(p12;q23) and DEK-NUP214/t(6;9)(p23;q24), probably due to appropriate classification to the HRG with HSCT in the first CR. However, an unsatisfactory outcome was still found in the patients with FLT3-ITD. This group of patients may benefit in the future from targeted therapy with FLT3 inhibitors that are currently widely being investigated.

Continued close international cooperation, further improvement of the stratification into risk groups and indications for HSCT based on genetics and MRD assessment, and new drugs (especially targeted treatment) seem to be crucial directions for the future.

## Figures and Tables

**Table 1:** The patient characteristics.

Protocol	AML-PPLLSG 83	AML-PPLLSG 94	AML-PPLLSG 98	AML-BFM 2004 Interim	AML-BFM 2012 Registry
Period	1983–1993	1994–1997	1998–2003	2004–2014	2015–2019
Follow-up end-point	31/03/2002	31/03/2002	31/07/2015	31/12/2020	31/12/2020
Median observation time (months)	128	93	119	90	37
Number of patients	187	74	151	356	131
Age median/range (years)	8.2/0.1–16.7	10.0/0.4–16.5	8.4/0–17.7	10.6/0–18	7.8/0.1–17.9
Gender male/female	93/94	36/38	87/64	184/172	73/58
CNS involvement	10 (5.3)	5 (6.7)	7 (5.8)	40 (11.2)	13 (9.9)
Extramedullary organ involvement	40 (21.4)	14 (18.9)	31 (20.5)	88 (24.7)	23 (17.5)
FAB types					
M0	0 (0)	3 (4.0)	12 (8.1)	28 (8.1)	2 (1,7)
M1	44 (23.5)	12 (16.2)	27 (18.2)	55 (16.0)	10 (8.3)
M2	54 (28.9)	24 (32.4)	52 (35.1)	94 (27.3)	36 (30.0)
M4	50 (26.7)	18 (24.3)	29 (19.6)	71 (20.6)	18 (15.0)
M5	30 (16.0)	11 (14.9)	19 (12.8)	70 (20.3)	39 (32.5)
M6	8 (4.3)	3 (4.0)	6 (4.1)	5 (1.4)	1 (8.3)
M7	1 (0.5)	1 (1.3)	3 (2.0)	21 (6.1)	14 (11.7)
Non defined	0 (0)	2 (2.7)	3 (2.0)	12 (3.5)	11 (9.2)
Risk groups					
SR	-	30 (40.6)	78 (51.7)	71 (19.9)	25 (18.5)
IR	-	-	-	-	52 (40.0)
HR	-	36 (48.6)	65 (43.0)	284 (79.8)	52 (40.0)
Unknown	-	8 (10.8)	8 (5.3)	1 (0.3)	2 (1.5)

**Table 2:** CNS treatment.

Protocol	AML-PPLLSG 83	AML-PPLLSG 94	AML-PPLLSG 98	AML-BFM 2004 Interim	AML-BFM 2012 Registry
**CNS irradiation**					
**Cycle**	Consolidation phase 2	SR Consolidation phase 2		SR and HR during maintenance	No prophylactic CNS irradiation
		HR short postremission block			
**Doses**	0–1 years 12 Gy, 1–2 years 15 Gy, >2 years 18 Gy	0-1 years 12 Gy, 1–2 years 15 Gy, >2 years 18 Gy	0–1 years no irradiation, 1–2 years 15 Gy, >2 years 18 Gy	<15 months no irradiation, 15 to <24 months 15 Gy, ≥24 months 18 Gy	No prophylactic CNS irradiation
**Intrathecal therapy**					
**Cycle**	consolidation phase 2 days 31, 38, 45, and 51	SR: consolidation phase 2 days 31, 38, 45, and 51		SR: AIE days 1 and 8, AI days 1 and 6, haM days 0 and 6, HAE day 0; maintenance treatment once a week for the first 4 weeks	cytarabine monotherapy: HAM, hAM, and HAE day 1 triple therapy (cytarabine, methotrexate, and prednizon): AIE days 1 and 8, AI or AI/2-CDA day 1, maintenance therapy days 1, 14, 28, and 42
		HR additional doses during AD and AE, and on days 1, 8, and 15 of a short postremission block		HR: additional dose HAM day 0	

CSN—central nervous system; SR—standard risk; HR—high risk. Cytarabine monotherapy: 0–1 year, 20 mg; 1–2 years, 26 mg; 2–3 years, 34 mg; >3 years, 40 mg. Triple therapy: cytarabine—0–1 year, 16 mg; 1–2 years, 20 mg; 2–3 years, 26 mg; >3 years, 30 mg; methotrexate—0–1 year, 6 mg; 1–2 years, 8 mg; 2–3 years, 10 mg; >3 years, 12 mg; prednizon—0–1 year, 4 mg; 1–2 years, 6 mg, 2–3 years, 8 mg; >3 years, 10 mg.

**Table 3:** Stratification to the risk groups.

Protocol	AML-PPLLSG 94	AML-PPLLSG 98	AML-BFM 2004 Interim	AML-BFM 2012 Registry
**SRG**	FAB other than M5 and <5% blasts in BM on day 15	FAB other than M5, <5% blasts in BM on day 15, no increase in blast count after day 15	M1/M2 with Auer rods ^a,b^ AML with t(8;21) ^a,b^ M4Eo with inv16 ^a,b^	t(8;21), inv(16), t(1;11), NPM1, CEBPαdm ^c^
**IRG**	-	-	-	All others ^d^
**HRG**	All other patients	All other patients	M0 M1/M2 without Auer rods M4, M5, M6 and M7	t(4;11), t(5;11), t(6;11), t(10;11), t(6;9), t(7;12), der12p, isolated monosomy 7, t(9;22), FLT3-ITD-WT1mut, complex karyotype

SRG—standard risk group; IRG—intermediate risk group; HRG—high risk group; BM—bone marrow; ^a^ In case of FLT3-ITD—reclassification to HRG; ^b^ In case of BM blasts >5% on day 15 or blastic reconstitution between days 15 and 28—reclassification to HRG; ^c^ In case of BM blasts >20% on the days 21−28—reclassification to IRG; ^d^ In case of BM blast > 20% on the days 21−28 or >5% on the days 42−56—reclassification to HRG.

**Table 4:** Hematopoietic stem cell transplantation (HSCT) in the consecutive protocols.

Protocol	AML-PPLLSG 83 *N* = 187	AML-PPLLSG 94 *N* = 74	AML-PPLLSG 98 *N* = 151	AML-BFM 2004 Interim *N* = 356	AML-BFM 2012 Registry *N* = 131
Indication for HSCT in the I CR	Very limited access	SRG and HRG: recommended but limited access	SRG: not recommended; HRG: recommended	Allo MSD HSCT: all HRG patients Allo MUD-HSCT: children with >5% of blasts or still in aplasia 4 weeks after second induction	Allo MSD/MUD HSCT: all HRG patients
Number of patients with indications (OS < 4 months and death without CR excluded)			52	245	43
HSCT in I CR (% of all patients/ % of patients with indications)	6 (3)	13 (18)	20 (13/38)	100 (28/41)	37 (28/86)
Auto-HSCT Allo-HSCT MSD (% of allo) MUD (% of allo) MMD (% of allo) Haplo (% of allo) No data	0 6 nd nd nd nd 6	5 8 nd nd nd nd 8	9 11 nd nd nd nd 11	1 99 50 (53) 20 (21 3 (3) 0 26	0 37 6 (16) 22 (59) 1 (3) 2 (5) 6

HSCT—hematopoietic stem cell transplantation; HRG—high risk group; SRG—standard risk group; MSD—matched sibling donor; MUD—matched unrelated donor; MD—mismatched donor; Haplo—HSCT from haploidentical donor; OS—overall survival; CR—complete morphological remission; Allo—allogeneic; Auto—autologous; nd—no data.

**Table 5:** Cytogenetics and molecular genetics.

Protocol	AML-BFM 2004 Interim *N* = 356	AML-BFM 2012 Registry *N* = 131
Fusion genes—number of results (%)	292 (82)	125 (95.4)
	Number of positive results (% of available results)	
RUNX1-RUNX1T1/t(8;21)(q22;q22)	51 (17.5)	16 (12.8)
CBFβ-MYH11/inv(16)(p13;q22)	12 (4.1)	12 (9.6)
All KMT2A rearangements	41 (14.0)	37 (29.6)
KMT2A-ELL/t(11;19)(q23;p13.3)	1 (0.3)	2 (1.6)
KMT2A-MLLT1/t(11;19)(q23;p13.3)	4 (1.4)	1 (0.8)
KMT2A-MLLT3/t(9;11)(p22;q23)	7 (2.4)	14 (11.2)
KMT2A-MLLT4/t(6;11)(q27;q23)	3 (1.0)	3 (2.4)
KMT2A-MLLT6/t(11;170(q23;q21)	4 (1.4)	0
KMT2A-MLLT10/t(10;11)(p12;q23)	7 (2.4)	16 (12.8)
KMT2A-AFF1/t(4;11)(q21;q23)	1 (0.3)	0
ETV6-MN1/t(12;22)(p13;q11)	0	1 (0.8)
DEK-NUP214 / t(6;9)(p23;q24)	3 (1.0)	5 (4.0)
BCR-ABL1/t(9;22)(q34;q11)	1 (0.3)	1 (0.8)
FLT3-ITD—number of results (%)	278 (78)	129 (98)
Positive (% of available)	30 (10.8)	11 (8.5)

**Table 6:** Treatment results in the five consecutive periods.

Protocol	AML-PPLLSG 83 *N* = 187	AML-PPLLSG 94 *N* = 74	AML-PPLLSG 98 *N* = 151	AML-BFM 2004 Interim *N* = 356	AML-BFM 2012 Registry *N* = 131
Complete morphological remission—CR (%)	133 (71)	49 (66)	127 (84)	310 (87)	114 (87)
Non-responders (%)	9 (5)	15 (20)	16 (11)	25 (7)	9 (7)
Early deaths (<42 days) (%)	45 (24)	10 (14)	8 (5)	21 (6)	8 (6)
Deaths in remission (%)	21 (11)	9 (12)	14 (9)	18 (5)	2 (1.5)
Relapses (%)	54 (29)	17 (23)	50 (33)	110 (31)	22 (17)
Continuous remission (%)	58 (31)	23 (31)	63 (42)	182 (51)	90 (69)
Probability of 3-years/5-years OS ± SD	0.34 ± 0.03/ 0.31 ± 0.03	0.37 ± 0.05/ 0.37 ± 0.05	0.54 ± 0.04/ 0.52 ± 0.04	0.67 ± 0.03/ 0.64 ± 0.03	0.75 ± 0.05/nd
Probability of 3-years/5-years EFS ± SD	0.31 ± 0.03/ 0.30 ± 0.03	0.34 ± 0.05/ 0.33 ± 0.05	0.44 ± 0.04/ 0.42 ± 0.04	0.53 ± 0.03/ 0.51 ± 0.03	0.67 ± 0.05/nd
Probability of 3-years/5-years RFS ± SD	0.52 ± 0.03/ 0.51 ± 0.03	0.65 ± 0.05/ 0.62 ± 0.05	0.58 ± 0.04/ 0.56 ± 0.04	0.66 ± 0.03/ 0.63 ± 0.03	0.78 ± 0.05/nd

nd—no data (because of the short follow-up in the last period (protocol AML-BFM 2012 Registry), only the probabilities of three-year survival were assessed). OS—overall survival; EFS—event-free survival; RFS—relapse free survival; SD—standard deviation.

**Table 7:** Prognostic factors: Cox regression univariate analysis (significant results).

Prognostic Factor: Survival Time	HR	95% CI	*p*
FLT3-ITD: EFS	2.37	1.59–3.53	0.000023
FLT3-ITD: RFS	2.65	1.58–4.44	0.000233
FLT3-ITD: OS	1.85	1.15–2.39	0.011539
WT1 mutation: EFS	3.14	1.44–6.88	0.004167
WT1 mutation: RFS	3.26	1.08–9.84	0.036058
WT1 mutation: OS	3.52	1.49–8.30	0.004035
RUNX1-RUNX1T1: OS	0.51	0.29–0.90	0.020423
WBC > 100,000/µL: EFS	1.82	1.38–2.40	0.000017
WBC > 100,000/µL: RFS	1.94	1.34–2.79	0.002167
WBC > 100,000/µL: OS	1.62	1.18–2.22	0.000363

HR—hazard ratio; CI—confidence interval; EFS—event free survival; RFS—relapse free survival; OS—overall survival; WBC—number of white blood cells at diagnosis.

## Data Availability

The data presented in this study are available upon request from the corresponding author. The data are not publicly available due to privacy and ethical restrictions.

## Supplementary Materials

The following are available online at https://www.mdpi.com/article/10.3390/cancers13184536/s1. Figure S1: Probability of survival in patients treated with AML-BFM 2004 Interim depending on the risk groups. Figure S2: Probability of survival in patients treated with AML-BFM 2012 Registry depending on the risk groups. Figure S3: Probability of survival depending on FLT3-ITD status in patients treated with AML-BFM 2004 Interim and AML-BFM 2012 Registry. Figure S4: Probability of overall survival (OS), event-free survival (EFS), and relapse free survival (RFS) in patients with CBFB-MYH11/inv(16)(p13;q22) treated with AML-BFM 2004 Interim and AML-BFM 2012 Registry. Figure S5: Probability of overall survival (OS), event-free survival (EFS), and relapse free survival (RFS) in patients with RUNX1-RUNX1T1 t(8;21)(q22;q22) treated with AML-BFM 2004 Interim and AML-BFM 2012 Registry. Figure S6: Probability of survival in patients with RUNX1-RUNX1T1 t(8;21)(q22;q22) depending on the treatment protocol AML-BFM 2004 Interim vs. AML-BFM 2012 Registry.
