# Supplementary material for: Advances in the First Line Treatment of Pediatric Acute Myeloid Leukemia in the Polish Pediatric Leukemia and Lymphoma Study Group from 1983 to 2019
Source: Cancers (Basel). 2021 Sep 9;13(18):4536. doi: 10.3390/cancers13184536 (PMC8472575; doi:10.3390/cancers13184536)
Supplement: Supplementary file 1 [file cancers-13-04536-s001.zip › cancers-1344375-SI.pdf]

Supplementary Materials: Advances in the First Line Treatment of Pediatric Acute Myeloid Leukemia in the Polish Pediatric Leukemia and Lymphoma Study Group from 1983 to 2019

Małgorzata Czogała, Walentyna Balwierz, Katarzyna Pawińska-Wąsikowska, Teofila Książek, Karolina Bukowska-Strakova, Wojciech Czogała, Barbara Sikorska-Fic, Michał Matysiak, Jolanta Skalska-Sadowska, Jacek Wachowiak, Małgorzata Moj-Hackemer, Krzysztof Kałwak, Katarzyna Muszyńska-Rosłan, Maryna Krawczuk-Rybak, Dominik Grabowski, Jerzy Kowalczyk, Lucyna Maciejka-Kembłowska, Ninela Irga-Jaworska, Katarzyna Bobeff, Wojciech Młynarski, Renata Tomaszewska, Tomasz Szczepański, Agnieszka Chodała-Grzywacz, Grażyna Karolczyk, Agnieszka Mizia-Malarz, Katarzyna Mycko, Wanda Badowska, Karolina Zielezińska, Tomasz Urasiński, Justyna Urbańska-Rakus, Małgorzata Ciebiera, Radosław Chaber, Natalia Bartoszewicz, Mariusz Wysocki and Szymon Skoczeń

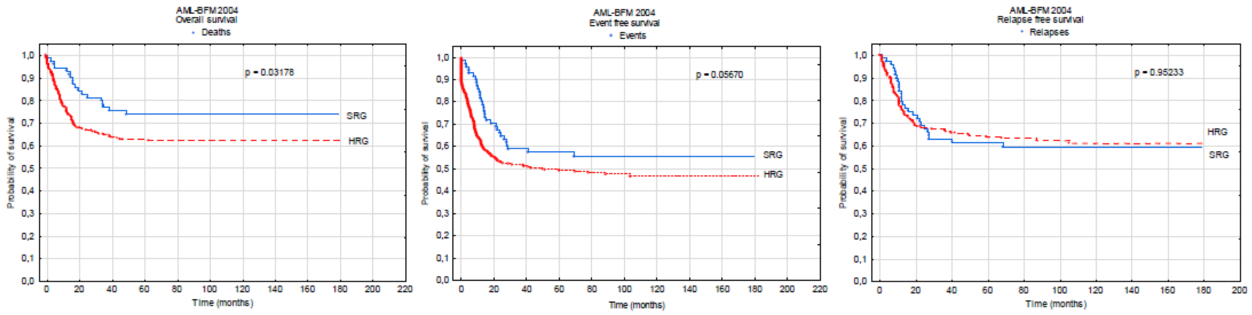

(a) **(b)** **(c)**

Figure S1. Probability of survival in patients treated with AML-BFM 2004 Interim depending on the risk groups. (**a**) Overall survival. (**b**) Event-free survival. (**c**) Relapse-free survival.

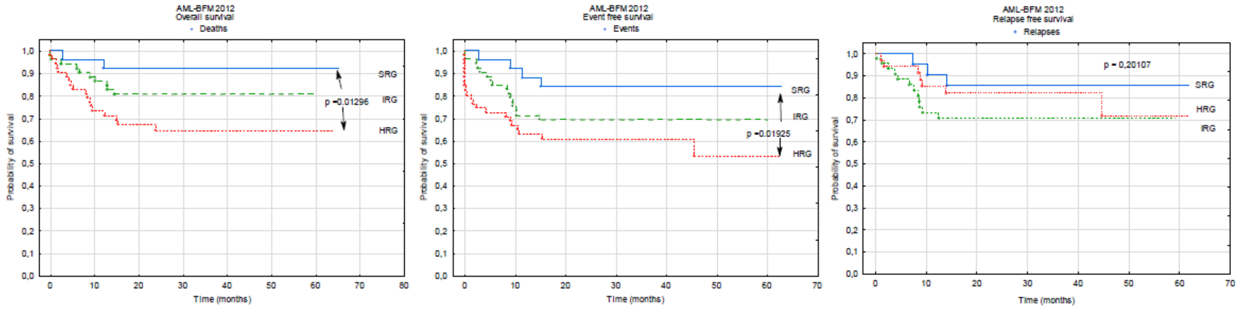

(a) **(b)** **(c)**

Figure S2. Probability of survival in patients treated with AML-BFM 2012 Registry depending on the risk groups. Statistically significant differences are marked. (**a**) Overall survival. (**b**) Event-free survival. (**c**) Relapse-free survival.

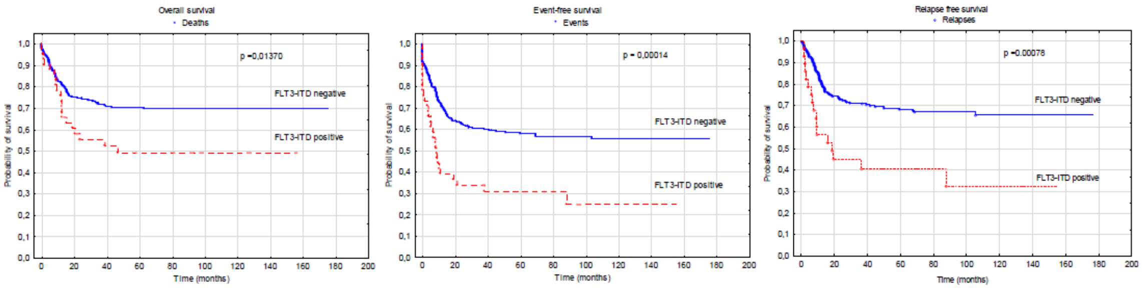

(a) **(b)** **(c)**

Figure S3. Probability of survival depending on FLT3-ITD status in patients treated with AML-BFM 2004 Interim and AML-BFM 2012 Registry. (**a**) Overall survival. (**b**) Event-free survival. (**c**) Relapse-free survival.

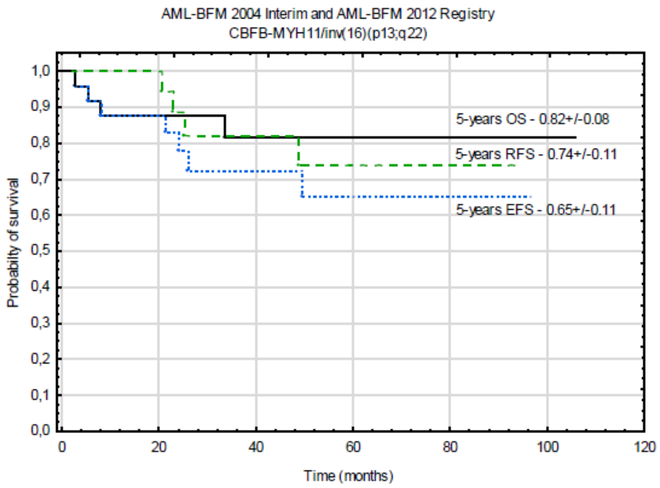

Figure S4. Probability of overall survival (OS), event-free survival (EFS) and relapse free survival (RFS) in patients with CBFB-MYH11/inv(16)(p13;q22) treated with AML-BFM 2004 Interim and AML-BFM 2012 Registry.

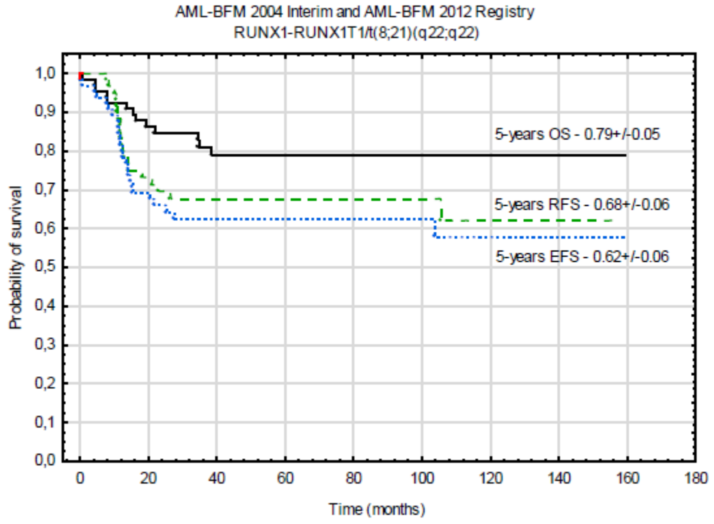

Figure S5. Probability of overall survival (OS), event-free survival (EFS) and relapse free survival (RFS) in patients with RUNX1-RUNX1T1 t(8;21)(q22;q22) treated with AML-BFM 2004 Interim and AML-BFM 2012 Registry.

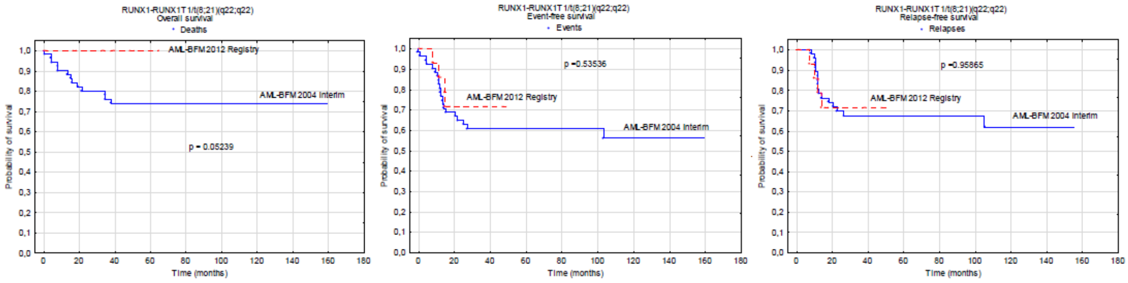

(a) (b) (c)

Figure S6. Probability of survival in patients with RUNX1-RUNX1T1 t(8;21)(q22;q22) depending on the treatment protocol AML-BFM 2004 Interim vs AML-BFM 2012 Registry.
